# Modeling protease-sensitive human pancreatic lipase mutations in the mouse ortholog

**DOI:** 10.1016/j.jbc.2024.107763

**Published:** 2024-09-10

**Authors:** Gyula Hoffka, Samara Mhana, Marcell Vas, Vanda Toldi, János András Mótyán, József Tőzsér, András Szabó

**Affiliations:** 1 Department of Biochemistry and Molecular Biology, Faculty of Medicine, University of Debrecen, Debrecen, Hungary; 2 Doctoral School of Molecular, Cell and Immune Biology, University of Debrecen, Debrecen, Hungary

**Keywords:** lipase degradation, proteolysis, enzyme mutation, trypsin, chymotrypsin, protein structure, molecular dynamics

## Abstract

Pancreatic lipase (PNLIP) is the major lipolytic enzyme secreted by the pancreas. A recent study identified human PNLIP variants P245A, I265R, F300L, S304F, and F314L in European cohorts with chronic pancreatitis. Functional analyses indicated that the variants were normally secreted but exhibited reduced stability when exposed to pancreatic proteases. Proteolysis of the PNLIP variants yielded an intact C-terminal domain, while the N-terminal domain was degraded. The protease-sensitive PNLIP phenotype was strongly correlated with chronic pancreatitis, suggesting a novel pathological pathway underlying the disease. To facilitate preclinical mouse modeling, here we investigated how the human mutations affected the secretion and proteolytic stability of mouse PNLIP. We found that variants I265R, F300L, S304F, and F314L were secreted at high levels, while P245A had a secretion defect and accumulated inside the cells. Proteolysis experiments indicated that wild-type mouse PNLIP was resistant to cleavage, while variant I265R was readily degraded by mouse trypsin and chymotrypsin C. Variants F300L, S304F, and F314L were unaffected by trypsin but were slowly proteolyzed by chymotrypsin C. The proteases degraded the N-terminal domain of variant I265R, leaving the C-terminal domain intact. Structural analyses suggested that changes in stabilizing interactions around the I265R mutation site contribute to the increased proteolytic susceptibility of this variant. The results demonstrate that variant I265R is the best candidate for modeling the protease-sensitive PNLIP phenotype in mice.

Human pancreatic lipase (PNLIP) is one of the most abundant enzymes secreted almost exclusively by the fully developed exocrine pancreas (1). It is responsible for hydrolyzing the majority of dietary triglycerides. The PNLIP protein comprises 465 amino acids, and of these, the first 16 residues at the N terminus form the secretory signal peptide (1). The molecular weight of the secreted enzyme excluding the signal peptide is about 50 kDa (2). PNLIP contains an α/β hydrolase fold N-terminal domain (residues 18–352), which carries the active site, and a beta sandwich fold C-terminal domain (residues 355–465) responsible for binding a cofactor protein, colipase (3). Bile acids can inactivate PNLIP after secretion into the small intestine (3). The primary function of colipase is to prevent the PNLIP protein from the inactivating effect of bile acids (3). PNLIP can exist in both inactive and active conformation. A hinge movement in a surface loop in the N-terminal domain configures the active site and activates the enzyme (4).

PNLIP may contribute to obesity by the hydrolysis of excessive amounts of dietary fats in the gut (5). PNLIP additionally facilitates the mobilization of toxic fatty acids from visceral fat in acute pancreatitis, which can exacerbate the severity of the condition (6, 7, 8). Mutations that diminish PNLIP secretion can lead to rare diseases such as pancreatic lipase deficiency and chronic pancreatitis (9, 10, 11, 12, 13). Notable among these is the homozygous T221M PNLIP mutation, which was described in two brothers of Arabic ancestry suffering from lipase deficiency (9). Functional analyses revealed that the T221M PNLIP variant promoted endoplasmic reticulum (ER) stress *via* protein misfolding and caused PNLIP protein accumulation as intracellular aggregates (10). Chronic pancreatitis frequently develops as a consequence of prolonged ER stress. Since the patients were diagnosed with pancreatic exocrine insufficiency, a symptom of chronic pancreatitis, they may also have developed mild forms of this disease (10). Genetically engineered mouse models provide a useful platform to validate mutations that contribute to chronic pancreatitis (14, 15, 16, 17, 18, 19, 20, 21, 22, 23). A mouse model of the T221M PNLIP variant confirmed its pathogenic nature in the disease (23).

Recently, rare heterozygous PNLIP mutations were reported in European chronic pancreatitis cohorts (24, 25). Consistent with previous observations, some variants exhibited reduced secretion caused by PNLIP misfolding, intracellular accumulation, and ER stress (26). In these cohorts, other PNLIP variants such as P245A, I265R, F300L, S304F, and F314L did not influence lipase secretion but were degraded by pancreatic proteases (24). The protease-sensitive variants exhibited a cumulative association with chronic pancreatitis, but the disease mechanism of these variants remained uncertain (24, 25). The number of mouse genetic models has recently increased, but only a limited number of studies have been performed using mouse lipase variants (20, 21, 23). Additionally, research has shown that the biochemical impacts of human mutations can vary between mouse and human pancreatic enzymes (27). In the present study, we introduced PNLIP mutations predisposing to proteolysis into the mouse PNLIP protein and assessed the secretion and proteolytic stability of these variants in the presence of mouse pancreatic proteases to assist in the development of PNLIP genetic mouse models.

## Results

### Structure of mouse and human PNLIP proteins

**Figure 1:**
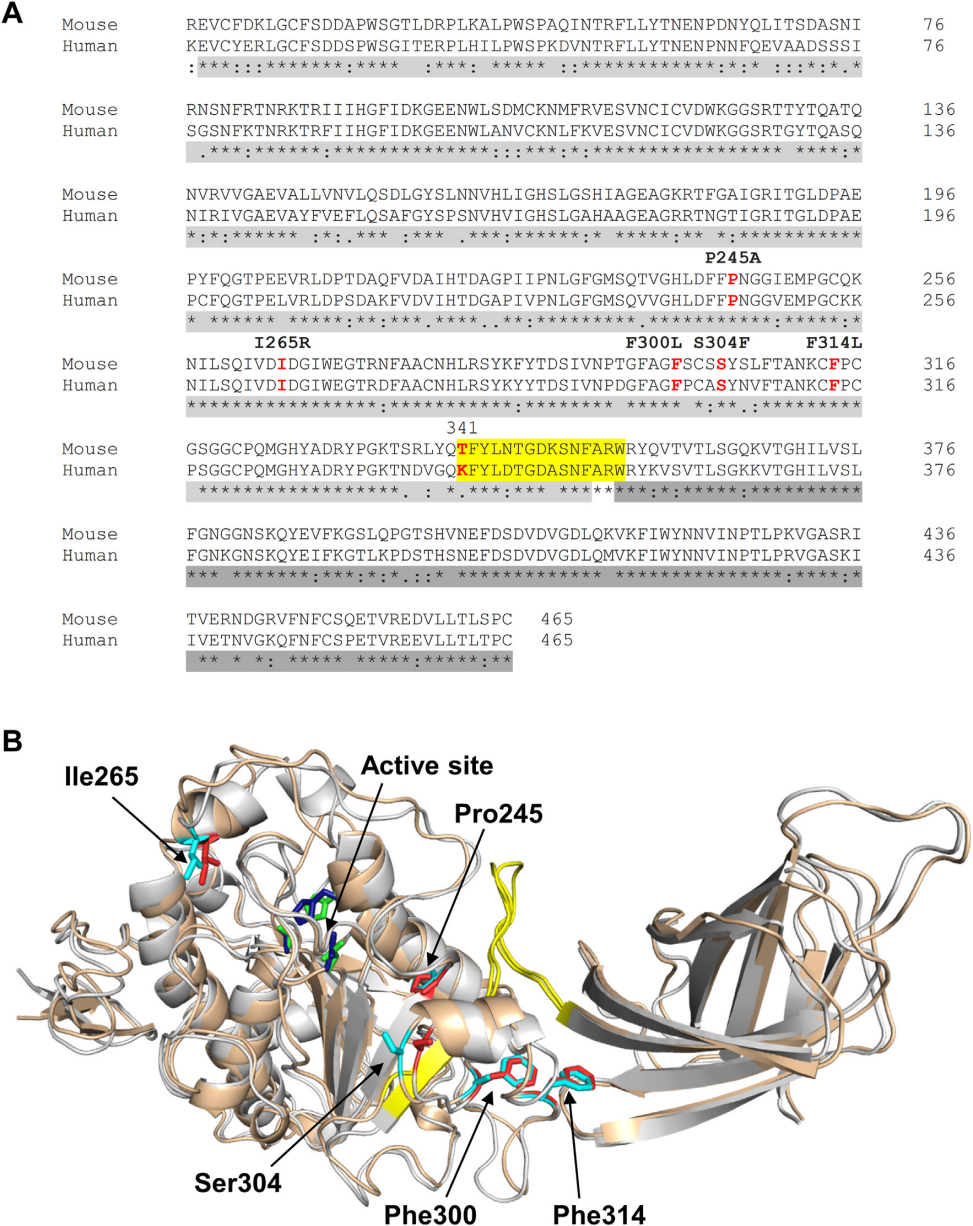
**Structural comparison of mouse and human PNLIP proteins.***A*, sequence alignment of mouse and human PNLIP proteins without the signal peptides. Chronic pancreatitis-associated mutation sites (Pro245, Ile265, Phe300, Ser304, and Phe314) are shown in red and the proteolysis-sensitive region with a *yellow* background. Note that the major tryptic cleavage site at Lys341 is not present in mouse PNLIP. *B*, ribbon models of mouse and human PNLIP. The mouse PNLIP is colored in *grey* and the human PNLIP is colored in wheat. The investigated mutation sites (Pro245, Ile265, Phe300, Ser304, and Phe314) are shown in red and light blue for mouse and human PNLIP, respectively. The N- and C-terminal domains and the proteolysis-sensitive region are highlighted in light *grey*, dark *grey*, and *yellow*, respectively.

Structural comparison revealed that both the human and the mouse PNLIP proteins without the signal peptide comprise 448 amino acid residues and share around 79% sequence identity (Fig. 1*A*). Additionally, mutation sites Pro245, Ile265, Phe300, Ser304, and Phe314 were found to be conserved in both proteins. Structural modeling demonstrated that 21.9% and 25.6% of the total residues formed alpha helices and beta sheets in mouse PNLIP, and 19.8% and 26.9% of the total residues contributed to alpha helices and beta sheets in human PNLIP, respectively (Fig. 1*B*). The root-mean-square deviation (RMSD) calculated between the two structures was 1.40 Å. Owing to the structural similarities, we considered the mouse PNLIP as a suitable candidate to study the biological effects of the mutations of the human ortholog identified in chronic pancreatitis.

### Secretion of mouse PNLIP variants from transfected cells

**Figure 2:**
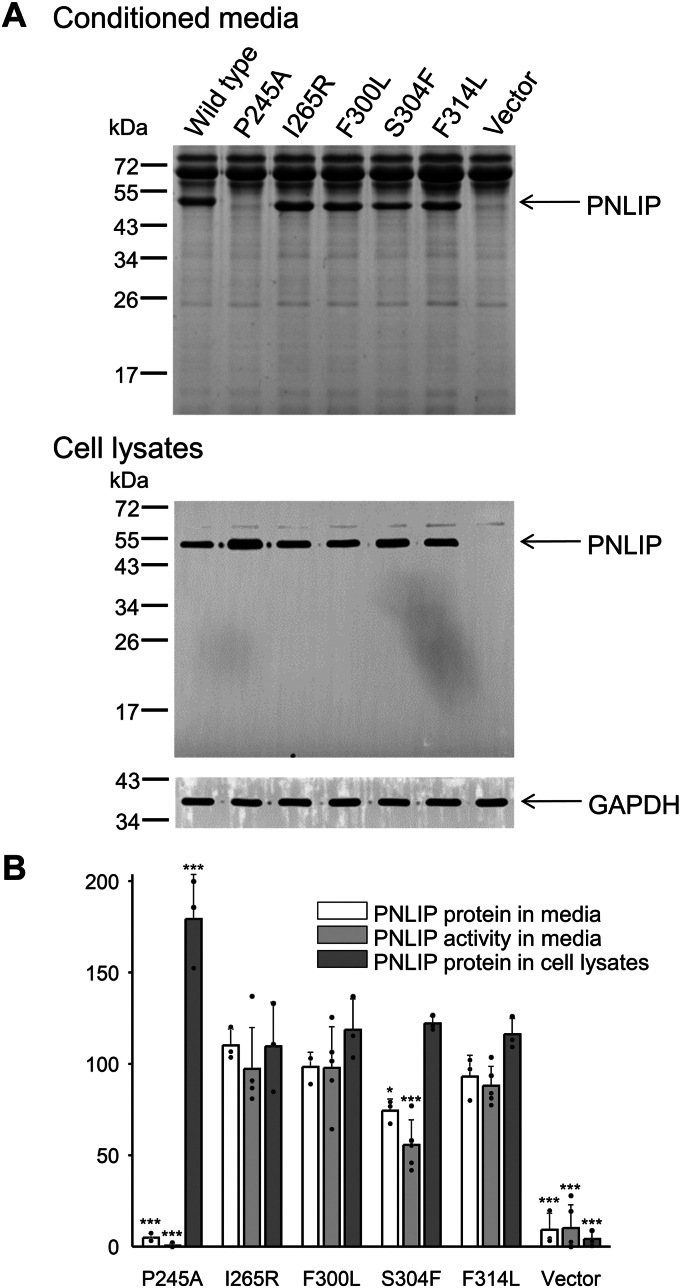
**Secretion of mouse PNLIP variants.***A*, PNLIP protein levels in the conditioned media of transiently transfected HEK 293T cells were analyzed by reducing SDS-PAGE and Coomassie staining (*upper* panel). PNLIP protein levels in the total cell lysates were determined by SDS-PAGE and western blotting (*lower* panel). The levels of GAPDH were also assessed as controls. *B*, densitometric evaluation of PNLIP band intensities in the conditioned media and cell lysates; and assessment of PNLIP enzyme activities in the conditioned media. Samples for all the experiments were collected 48 h after transfection. Mean ± SD of 3 to 5 independent biological replicates are shown. Note that some data points exhibit overlap. ∗*p* ≤ 0.05; ∗∗∗*p* ≤ 0.001.

To study the effect of PNLIP mutations on the secretion of mouse PNLIP, we transiently transfected HEK 293T cells with wild-type and mutant PNLIP plasmids and determined the levels of PNLIP in both the conditioned media and cell lysates (Fig. 2). The results indicated that all the variants are expressed. Secretion of the I265R, F300L, and F314L PNLIP variants was comparable to that of the wild type, whereas secretion of the S304F variant was slightly reduced. Lipase activity measurements of the conditioned media indicated that all of these variants exhibited full enzyme activity. Both the secretion and activity assays confirmed that the PNLIP variants are properly folded. In contrast, the P245A PNLIP variant was not present in the medium but accumulated in the cells, probably because of protein misfolding. Based on these findings, we excluded the P245A variant from further studies.

### Proteolysis of mouse PNLIP variants by trypsin

**Figure 3:**
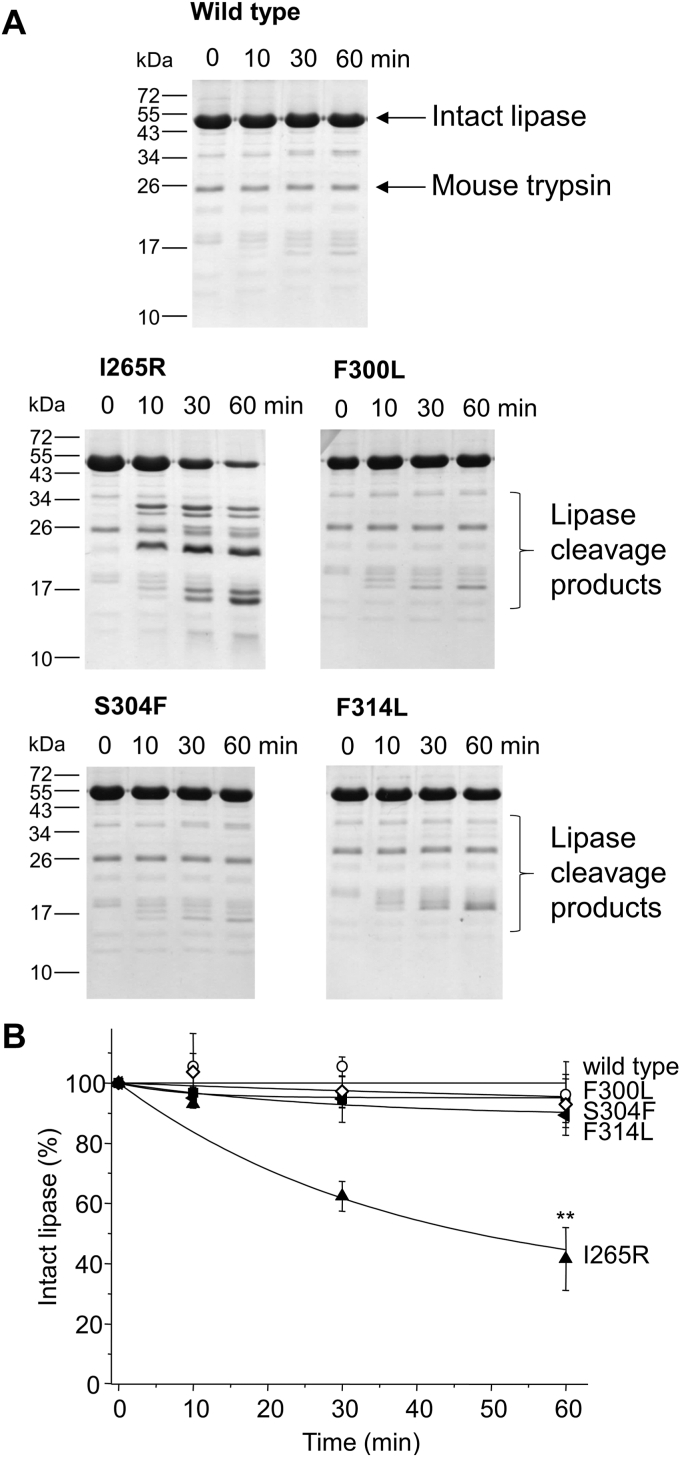
**Effect of mouse cationic T7 trypsin on mouse PNLIP variants.** Purified wild-type and mutant PNLIP proteins at a final concentration of 2 μM were incubated at 37 °C with 200 nM mouse T7 trypsin in 0.1 M Tris-HCl (pH 8.0), 50 mM NaCl, and 1 mM CaCl_2_ (final concentrations). At the indicated times, 75 μl aliquots were precipitated with 10% trichloroacetic acid (final concentration) and analyzed by reducing SDS-PAGE and Coomassie Blue staining. *A*, Representative gels of three experiments are shown. *B*, representative evaluation of PNLIP band intensities. Mean ± SD of three experiments are shown. Statistical significance relative to the wild-type value was calculated at 60 min ∗∗*p* ≤ 0.01.

**Figure 4:**
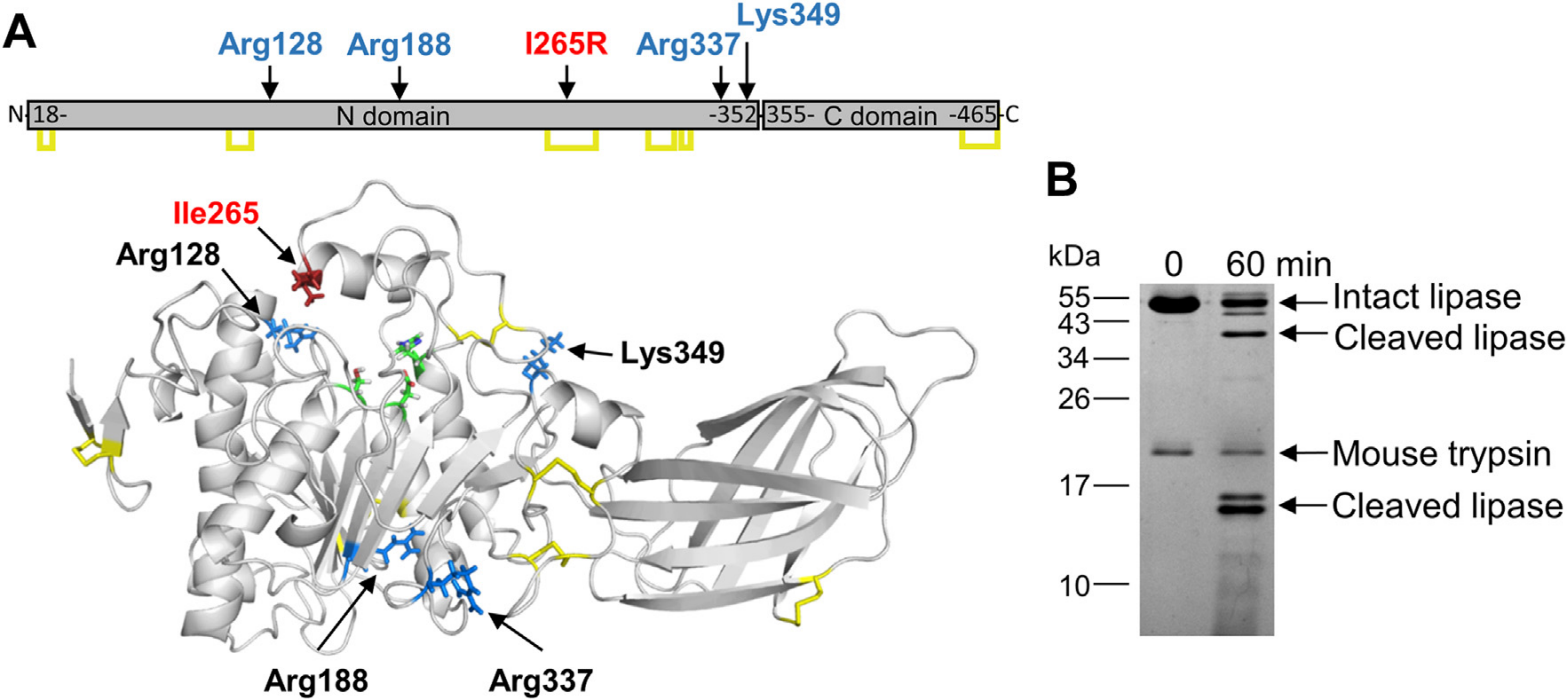
**Proteolytic cleavages in I265R mouse PNLIP variant by mouse T7 trypsin.***A*, structural model of mouse PNLIP. The model was generated as detailed in Experimental procedures. Residues in *blue* indicate cleavage sites identified in the PNLIP variant by N-terminal sequencing of major cleavage fragments. The mutation site, disulfide bonds, and active site catalytic triad are marked in *red*, *yellow*, and *green*, respectively. *B*, proteolytic degradation of the I265R mouse PNLIP variant. The mutant PNLIP protein (2 μM) was incubated at 37 °C with 200 nM mouse T7 trypsin in 0.1 M Tris-HCl (pH 8.0), and 1 mM CaCl_2_ (final concentrations). At the indicated times, 75 μl aliquots were precipitated with 10% trichloroacetic acid (final concentration) and analyzed by non-reducing SDS-PAGE and Coomassie Blue staining.

To study how these mutations affect the proteolytic stability of mouse PNLIP, we purified and assayed the wild-type PNLIP and variants with mouse cationic T7 trypsin in a 10:1 M ratio and followed PNLIP digestion over time using SDS-PAGE and Coomassie staining (Fig. 3). We observed that the wild-type PNLIP was resistant to proteolysis in the investigated 60-min interval. Interestingly, the F300L, S304F, and F314L variants remained mainly intact in the presence of T7 trypsin. Substantial proteolytic digestion by T7 trypsin was observed only for the I265R variant, resulting in several cleavage products migrating in the 15 to 30 kDa range. The proteolytic digestion of the I265R variant by T7 trypsin was also studied in the presence of colipase (Fig. S1). Interestingly, neither the digestion rate nor the banding pattern generated by trypsin was affected by colipase. The major protein bands of the I265R PNLIP variant generated by mouse T7 trypsin were subjected to N-terminal sequencing by Edman degradation. Figure 4*A* demonstrates the cleavage sites gathered from the N-terminal sequencing data. Cleavages at Arg128, Arg188, Arg337, and Lys349 were identified mostly in surface loops of the N-terminal domain of the PNLIP protein, while the C-terminal domain remained intact. The proteolytic degradation of the I265R mouse PNLIP variant by mouse T7 trypsin was also studied by non-reducing SDS-PAGE after performing the digestion experiment (Fig. 4*B*). The results indicated that the I265R mouse PNLIP variant was disintegrated into protein fragments during the incubation with trypsin. We assumed that mouse trypsin might have lower proteolytic activity than human trypsin; therefore, we repeated the digestion experiments with human cationic trypsin (Fig. S2). In agreement with our previous findings, only the I265R variant was readily digested with human trypsin, while the F300L, S304F, and F314L variants, together with the wild-type PNLIP, remained largely resistant to degradation.

### Proteolysis of mouse PNLIP variants by chymotrypsin

**Figure 5:**
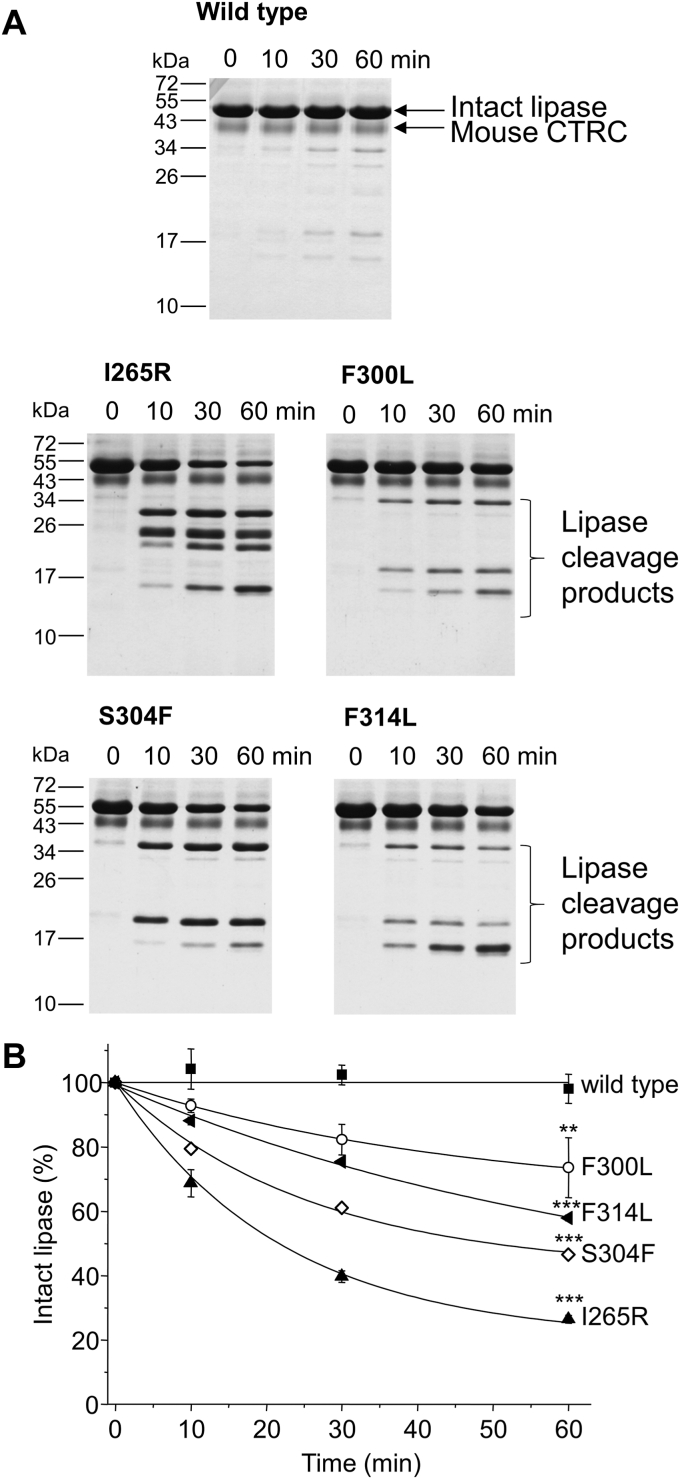
**Effect of mouse chymotrypsin C (CTRC) on mouse PNLIP variants.** Purified wild-type and mutant PNLIP proteins at a final concentration of 2 μM were incubated at 37 °C with 200 nM mouse CTRC in 0.1 M Tris-HCl (pH 8.0), 50 mM NaCl, and 1 mM CaCl_2_ (final concentrations). At the indicated times, 75 μl aliquots were precipitated with 10% trichloroacetic acid (final concentration) and analyzed by reducing SDS-PAGE and Coomassie Blue staining. *A*, representative gels of three experiments are shown. *B*, representative evaluation of PNLIP band intensities. Mean ± SD of three experiments are shown. Statistical significance relative to the wild-type value was calculated at 60 min ∗∗*p* ≤ 0.01; ∗∗∗*p* ≤ 0.001.

**Figure 6:**
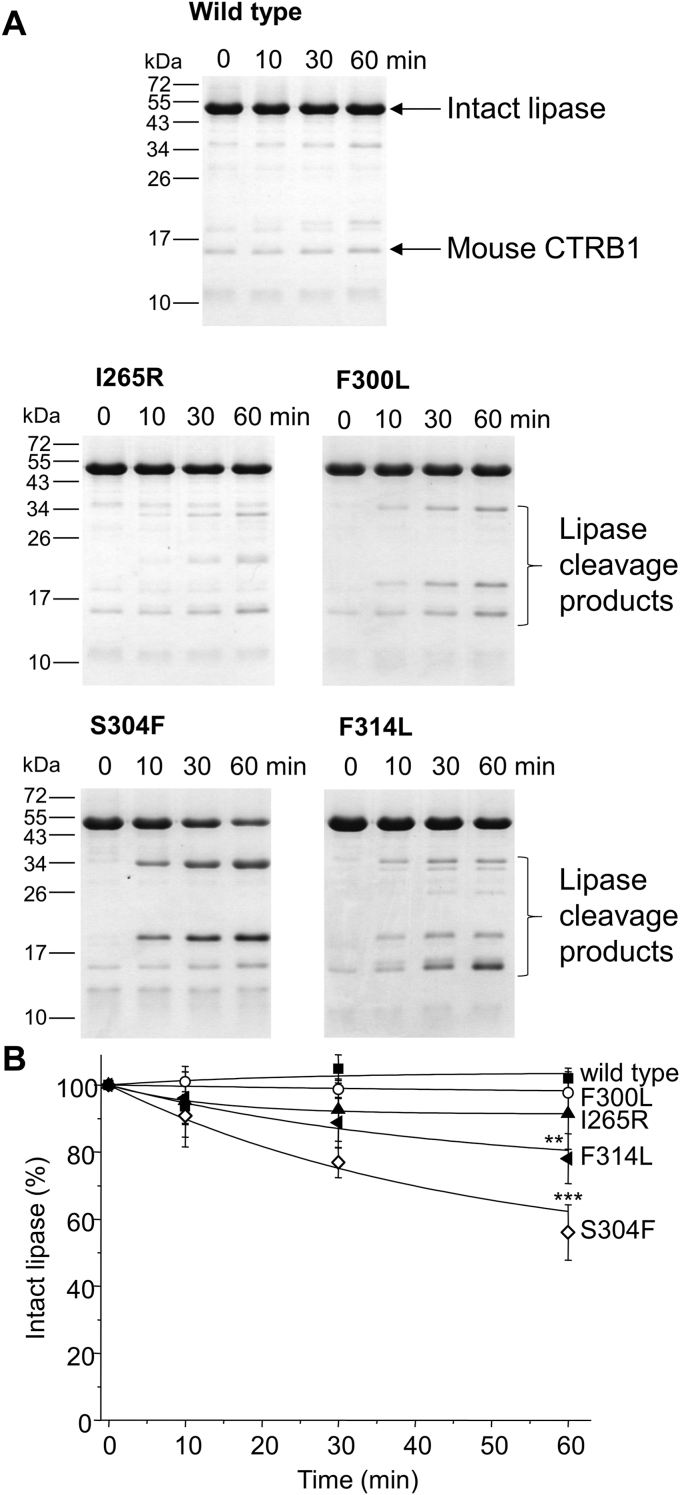
**Effect of mouse chymotrypsin B1 (CTRB1) on mouse PNLIP variants.** Purified wild-type and mutant PNLIP proteins at a final concentration of 2 μM were incubated at 37 °C with 200 nM mouse CTRB1 in 0.1 M Tris-HCl (pH 8.0), 50 mM NaCl, and 1 mM CaCl_2_ (final concentrations). At the indicated times, 75 μl aliquots were precipitated with 10% trichloroacetic acid (final concentration) and analyzed by reducing SDS-PAGE and Coomassie Blue staining. *A*, representative gels of three experiments are shown. *B*, representative evaluation of PNLIP band intensities. Mean ± SD of three experiments are shown. Statistical significance relative to the wild-type value was calculated at 60 min ∗∗*p* ≤ 0.01; ∗∗∗*p* ≤ 0.001.

**Figure 7:**
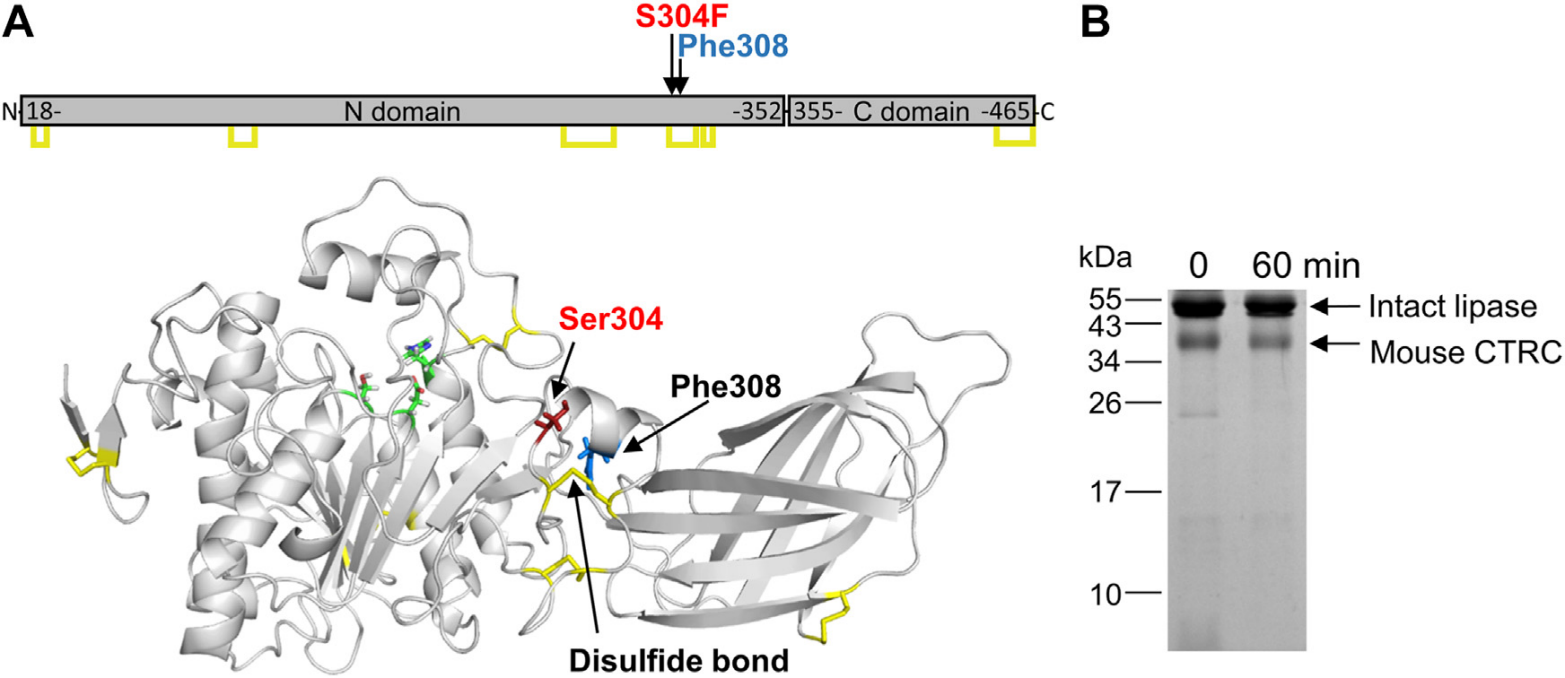
**Proteolytic cleavage in S304F mouse PNLIP variant by mouse CTRC.***A*, structural model of mouse PNLIP. The model was generated as described in Experimental Procedures. Residue in *blue* indicates a cleavage site identified in the PNLIP variant by N-terminal sequencing of the major cleavage fragments migrating at 15–20 kDa. The mutation site, disulfide bonds, and active site catalytic triad are marked in *red*, *yellow*, and *green*, respectively. *B*, proteolytic degradation of the S304F mouse PNLIP variant. The mutant PNLIP protein (2 μM) was incubated at 37 °C with 200 nM mouse CTRC in 0.1 M Tris-HCl (pH 8.0), and 1 mM CaCl_2_ (final concentrations). At the indicated times, 75 μl aliquots were precipitated with 10% trichloroacetic acid (final concentration) and analyzed by non-reducing SDS-PAGE and Coomassie Blue staining.

To study the effect of other mouse pancreatic proteases on the stability of mouse PNLIP, we assayed the wild-type PNLIP and variants with mouse CTRC and CTRB1 and followed PNLIP degradation by SDS-PAGE and Coomassie staining (Figs. 5 and 6). Wild-type PNLIP remained remarkably stable in the presence of both CTRC and CTRB1. CTRC cleaved the PNLIP variants more readily than trypsin. The F300L, S304F, and F314L PNLIP variants were degraded moderately by CTRC, leading to three cleavage products migrating at approximately 36 kDa, 20 kDa, and 15 kDa. In comparison, somewhat quicker digestion of the I265R variant was observed by CTRC, resulting in four cleavage products migrating in the 15 to 30 kDa range. N-terminal sequencing of the degradation products of the S304F variant at 15 kDa and 20 kDa revealed a single cleavage site in a short α-helix of the N-terminal domain at Phe308 (Fig. 7*A*). Structural modeling revealed that this protein segment is stabilized by a disulfide bond, which may protect the PNLIP from degradation. Consistent with this notion, the analysis of the degradation products by non-reducing SDS-PAGE indicated that the cleavage of the S304F variant at Phe308 does not result in the disintegration of PNLIP (Fig. 7*B*). CTRB1 mostly cleaved the S304F variant, which was noticeably slower than the cleavage rate by CTRC. Interestingly, degradation of the I265R, F300L, and F314L PNLIP variants was ineffective by CTRB1. The cleavage pattern of the S304F variant by CTRB1 was identical to that of CTRC.

### Structural and dynamic analyses of mouse and human PNLIP

**Figure 8:**
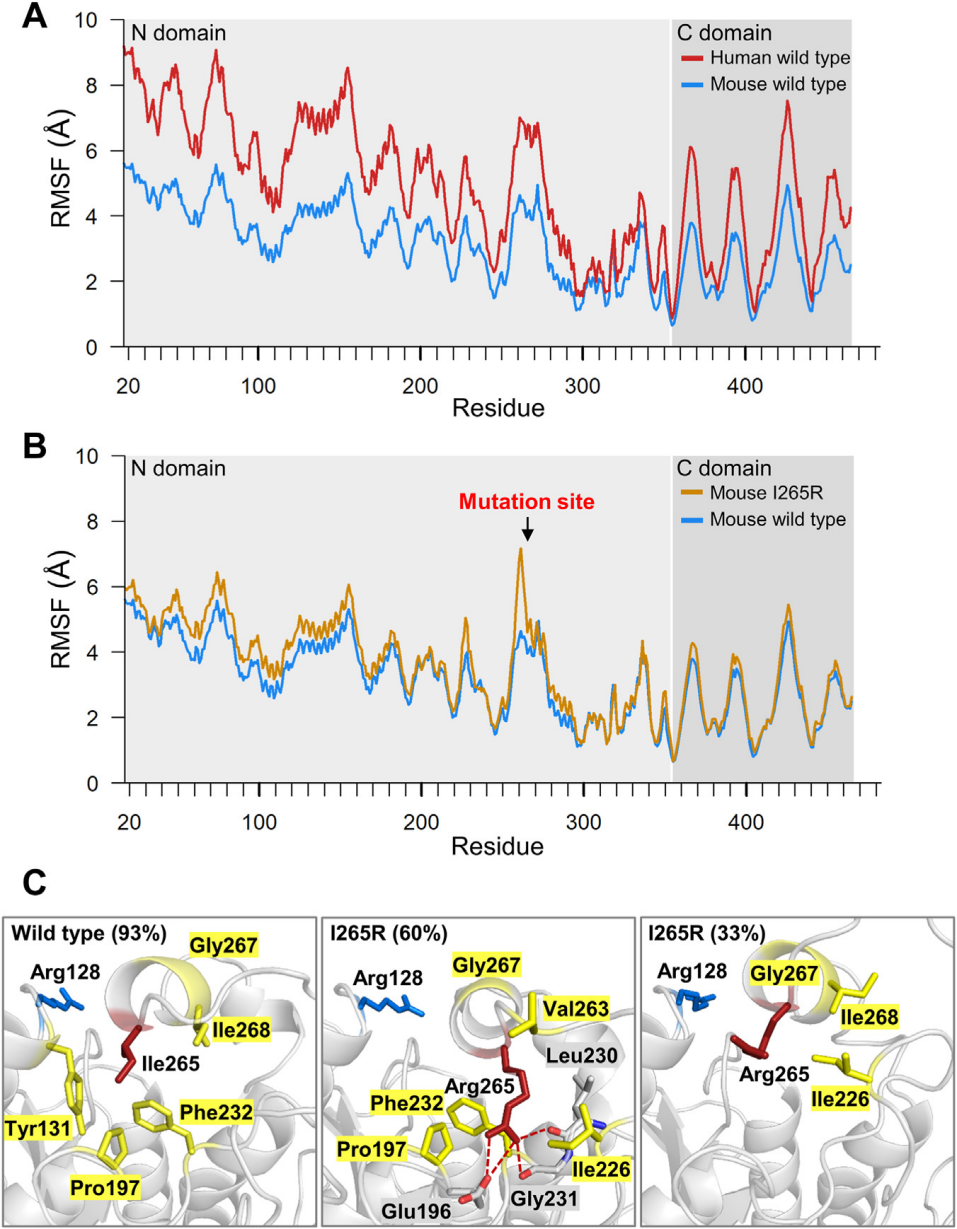
**Structural analyses of mouse wild-type PNLIP and I265R variant.** The Root-Mean-Square-Fluctuation (RMSF) values calculated for the Cα atoms of (*A*) mouse and human wild-type PNLIP proteins, and (*B*) mouse wild-type PNLIP and I265R variant, are presented as described in the Experimental Procedures. *C*, residues that interact with the Ile265 in the mouse wild-type PNLIP and the Arg265 in the mouse I265R variant were analyzed. The wild-type PNLIP and the I265R variant exhibit one and two primary clusters, respectively. Numbers in parentheses indicate the percentage of total frames corresponding to each specific cluster. Yellow residues represent apolar interactions with the Ile265 or Arg265 residues shown in *red*. Hydrogen bonds are labeled with *red* dashed lines. The tryptic cleavage site Arg128 is shown in *blue*.

**Figure 9:**
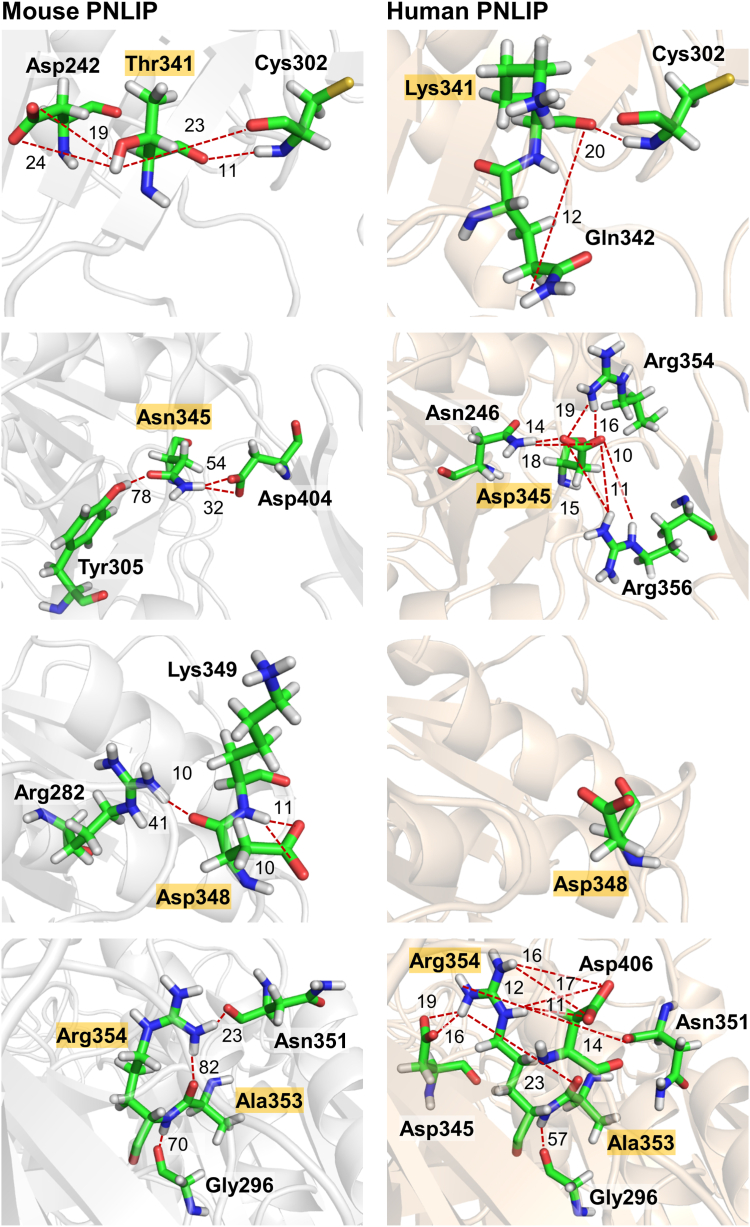
**Differences in hydrogen bond interactions formed by residues 341, 345, 348, 353, and 354 in mouse and human PNLIP.** Hydrogen bond interactions that occur in over 10% in the trajectories are shown (see Table S1 for details). Hydrogen bonds are highlighted with *red* dashed lines. The numbers indicate the occurrence of individual hydrogen bonds during the molecular dynamics trajectory, represented as a percentage.

Proteolytic degradation of the mouse PNLIP variants was substantially slower than that of the human PNLIP variants described earlier (24). To gain insight into the structural background of the observed differences, we performed molecular dynamics simulations and examined the structural changes and flexibility of the amino acid residues in both the mouse and human PNLIP proteins (Fig. 8). Analysis of the root-mean-square fluctuation (RMSF) values of the Cα atoms revealed relatively high flexibility for both proteins. Nevertheless, the RMSF values of the Cα atoms were found to be lower in the mouse compared to the human PNLIP (Fig. 8*A*). The results suggest that the mouse PNLIP protein possesses a more rigid structure than its human counterpart. RMSF analysis of the wild-type and I265R mouse PNLIP revealed increased flexibility of the Cα atoms in the vicinity of the mutation site (Fig. 8*B*). Structural analyses further demonstrated that the wild-type protein exhibits a single predominant cluster, whereas the I265R variant forms two main distinct clusters, representing 60% and 33% of the total frames (Fig. 8*C*). Subsequent analyses revealed that the Ile265 to Arg mutation transitions local interactions from apolar contacts to hydrogen bonding within the primary cluster and reduces overall interactions in the secondary cluster (Fig. 8*C*). These structural changes could enhance the accessibility and proteolytic susceptibility of Arg128 and other nearby tryptic cleavage sites. Proteolytic cleavage by pancreatic proteases that separates the N- and C-terminal domains of human PNLIP occurs within the 341 to 355 region (24). The hydrogen bond interaction of this region in the mouse and human PNLIP proteins were also compared. Substantial differences in hydrogen bond interactions and in their prevalence were observed at residues 341, 345, 348, 353, and 354 (Fig. 9 and Table S1). The analyses revealed that all these residues in mouse PNLIP are capable of forming substantially stronger and more prevalent hydrogen bond interactions compared to the corresponding residues in human PNLIP. Consequently, the region within the mouse PNLIP protein exhibits a more stable structure compared to the human protein.

### Effect of the T341K mutation on the proteolysis of mouse PNLIP variants by trypsin

**Figure 10:**
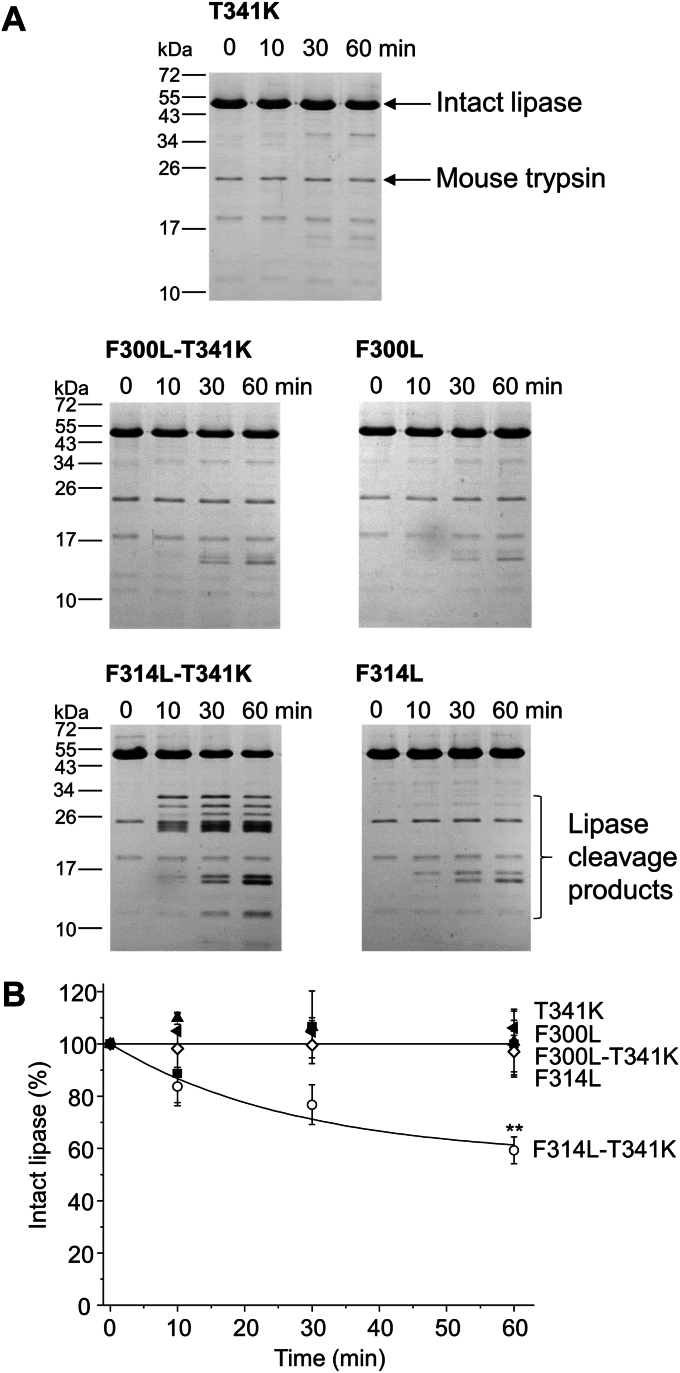
**Effect of the T341K mutation on the degradation of mouse PNLIP variants by mouse T7 trypsin.** Purified wild-type and mutant PNLIP proteins at a final concentration of 2 μM were incubated at 37 °C with 200 nM mouse T7 trypsin in 0.1 M Tris-HCl (pH 8.0), 50 mM NaCl, and 1 mM CaCl_2_ (final concentrations). At the indicated times, 75 μl aliquots were precipitated with 10% trichloroacetic acid (final concentration) and analyzed by reducing SDS-PAGE and Coomassie Blue staining. *A*, representative gels of three experiments are shown. *B*, representative evaluation of PNLIP band intensities. Mean ± SD of three experiments are shown. Statistical significance relative to the wild-type value was calculated at 60 min ∗∗*p* ≤ 0.01.

The major tryptic cleavage site in human PNLIP at position 341 is missing in mouse PNLIP (Fig. 1*A*). To study the significance of this site, we expressed and purified T341K, F300L-T341K, and F314L-T341K mouse PNLIP variants and studied their proteolytic stability in the presence of mouse T7 trypsin (Fig. 10). Interestingly, the T341K mutation, alone or in combination with the F300L mutation, resulted in no significant improvement in mouse PNLIP proteolysis. However, the T341K mutation, in combination with the F314L mutation, markedly increased the degradation of mouse PNLIP by T7 trypsin. The fragmentation pattern of the cleaved F314L-T341K mouse PNLIP variant was similar to that of the I265R variant (Figs. 10 and 3).

## Discussion

Growing evidence indicates that chronic pancreatitis often develops due to genetic susceptibility (28). Mutations in secretory proteins of the exocrine pancreas can lead to chronic pancreatitis through increased intrapancreatic protease activation or protein misfolding, intracellular retention, and cell death (29, 30, 31, 32). Recently, novel human PNLIP variants (P245A, I265R, F300L, S304F, and F314L) have been reported (24). These variants were readily secreted in transfected mammalian cells but were prone to proteolysis by pancreatic trypsin and chymotrypsin. The protease-sensitive phenotype was strongly associated with chronic pancreatitis, although the disease mechanism appeared unrelated to protein misfolding or trypsinogen activation. Nonetheless, a recent study found that the F300L human variant, which exhibited slightly reduced secretion in transfected mammalian cells, led to PNLIP intracellular accumulation and proteotoxicity (33).

In the present work, we introduced the P245A, I265R, F300L, S304F, and F314L mutations into mouse PNLIP and studied the secretion of these variants in transiently transfected HEK 293T cells. Secretion of the I265R, F300L, and F314L PNLIP variants was comparable to that of the wild type, whereas secretion of the S304F variant was slightly reduced. None of the variants accumulated intracellularly. Surprisingly, the P245A variant was not secreted; instead, it accumulated substantially in the cells. These data suggest that a severe reduction in mouse PNLIP secretion is required for its intracellular accumulation. Slightly reduced secretion of the mouse PNLIP variant did not result in intracellular accumulation, which contradicted previously published results on human variants (33).

We further examined how the P245A, I265R, F300L, S304F, and F314L mutations affected the proteolytic stability of mouse PNLIP in the presence of mouse cationic T7 trypsin, CTRC, and CTRB1 pancreatic proteases. As the P245A mouse PNLIP variant was not secreted, it was omitted from these experiments. Four of the 11 potentially functional mouse trypsin genes are expressed in the pancreas, of which T7 trypsin is one of the most abundant proteases. The mouse pancreas also synthesizes chymotrypsins of which CTRC is a minor isoform and CTRB1 is a major isoform (27). CTRC is known to regulate the activation of other pancreatic proteases through proteolytic cleavages, while CTRB1 functions as a highly effective digestive protease in the intestine (34, 35). The results demonstrated that the mouse PNLIP variants remained markedly stable in the presence of both mouse and human trypsins, except for the I265R variant, which was digested like its human counterpart. Proteolytic cleavage of the I265R variant occurred at multiple sites within the N-terminal domain, while the C-terminal domain remained unaffected. Structural analyses indicated that the I265R mutation induced structural changes in the surrounding region, which may result in enhanced proteolysis. The PNLIP protein contains six evolutionarily conserved disulfide bonds, five of which are located in the N-terminal domain. In the I265R variant, the polypeptide chains between the tryptic cleavage sites Arg128 and Arg188 and between Arg337 and Lys349 are not stabilized by disulfide bonds, which led to the disintegration of the N-terminal domain and inactivation of the enzyme during proteolysis. The analysis of the gel banding patterns, combined with the N-terminal sequencing data, suggests that the I265R mouse PNLIP variant is first cleaved at multiple sites within the 128 to 337 region, followed by additional cleavages in the 341 to 355 region. The I265R mouse PNLIP variant was also readily digested by CTRC. In addition, CTRC cleaved the F300L, S304F, and F314L variants, but at slower rates. Interestingly, CTRB1 only cleaved the S304F variant at an appreciable rate. CTRB1 primarily cleaves after Phe, Tyr, and Trp residues, while CTRC exhibits a broader substrate specificity, also cleaving after aliphatic residues such as Met and Leu (35). The differences in the substrate specificity between CTRB1 and CTRC may account for the increased proteolytic activity of CTRC on PNLIP variants. Digestion of the I265R PNLIP variant by chymotrypsin resulted in the disintegration of the PNLIP N-terminal domain, while leaving the C-terminal domain intact. In the case of the S304F PNLIP variant, only one major cleavage event occurred within a PNLIP segment stabilized by a disulfide bond. This prevented the separation of the protein domains. The chymotrypsin-mediated cleavage patterns of the F300L and F314L PNLIP variants were identical to that observed for the S304F variant.

Trypsin is a highly P1 site-specific protease (Schechter and Berger nomenclature) that cleaves peptide bonds in polypeptide chains after Arg and Lys residues. The major tryptic cleavage site at position 341 is missing from mouse PNLIP due to an evolutionary mutation. In an attempt to increase the proteolysis of mouse PNLIP, we mutated Thr341 to Lys in the wild-type, F300L, and F314L mouse PNLIP proteins. A noticeable increase in PNLIP cleavage by mouse trypsin was detected only for the F314L-T341K variant. These results suggest that the mouse PNLIP structure is inherently more stable than that of its human counterpart. To validate this assumption, we compared the flexibilities of the mouse and human PNLIP residues based on molecular dynamics simulations. Analysis of the RMSF of the Cα atoms showed that the mouse PNLIP protein exhibited significantly lower flexibility compared to the human PNLIP. Furthermore, we examined hydrogen bond interactions within the proteolysis-sensitive 341 to 355 linker region of the PNLIP N-terminal domain. The findings indicated more stable hydrogen bonds in mouse PNLIP compared to the human enzyme. These results confirmed that stabilizing interactions occur more frequently in the mouse PNLIP, accounting for its enhanced proteolytic stability.

Taken together, we performed functional experiments and *in silico* analyses to investigate the effects of chronic pancreatitis-associated human mutations on mouse PNLIP. The results indicated that the majority of readily secreted PNLIP variants were markedly more resistant to proteolysis than observed in the case of human PNLIP variants. Our results demonstrated that the I265R mouse PNLIP variant is the most suitable for studying the protease-sensitive phenotype in a mouse model.

## Experimental procedures

### Plasmid construction, mutagenesis, and nomenclature

Coding DNA for mouse PNLIP was cloned from C57BL/6J mouse pancreatic cDNA using the 5′-CTA GAC TCG AGG TCC GTA GAA CCT GAC GGA TCC-3′ forward primer and the 5′-ACT TAA GCT TAC AAC AGT GGG ACT TGG TGG TCA C-3′ reverse primer, where the XhoI and HindIII sites are underlined, respectively. The PCR amplicon was ligated into the pcDNA3.1(−) mammalian expression plasmid using XhoI and HindIII restriction sites. The last 192 bp of the mouse PNLIP cDNA was custom synthesized, carrying a coding DNA for a C-terminal 10 histidine tag, and cloned into the mouse PNLIP expression plasmid using EcoRI and HindIII restriction enzymes. The mammalian pcDNA3.1(−) expression plasmids harboring the coding DNA for mouse chymotrypsinogen C (CTRC) and chymotrypsinogen B1 (CTRB1) with C-terminal histidine tags have been described previously (27). Construction of the pTrapT7 plasmids harboring the coding DNA of human cationic trypsinogen and mouse T7 trypsinogen has been reported previously (27, 36). PNLIP mutations were introduced using overlap extension PCR mutagenesis and the final PCR products were ligated into the expression plasmid. The sequence of all PNLIP constructs was verified by capillary sequencing. The amino acid numbering of the PNLIP protein started with the translation initiator methionine.

### Cell culture, transfection, and cell lysis

Human Embryonic Kidney (HEK) 293T cells (Merck) were cultured and grown overnight in 2 ml of high-glucose DMEM cell culture media containing 10% FBS, 4 mM glutamine, 50 U/ml penicillin, and 50 μg/ml streptomycin in a 6-well tissue culture plate (1.2 million/well). At 80 to 90% confluence, cells were transiently transfected with 4 μg plasmid DNAs carrying wild-type and mutant PNLIP using branched chain polyethyleneimine transfection reagent as described previously (26, 37). After 6 h of incubation, the cells were rinsed with PBS and incubated in 2 ml of Opti-MEM I medium (Gibco) at 37 °C in a standard cell culture incubator. The conditioned medium was harvested 48 h after a medium change, and total protein was isolated from the cells with 0.2 ml Reporter Lysis 5× buffer (Promega) by freeze–thaw cycles. The insoluble particles were removed by centrifugation at 3500*g* for 5 min. The total protein concentration of the supernatant was determined using the Pierce BCA Protein Assay kit (Thermo Scientific).

### Lipase activity assay

Lipase activity in the conditioned media was measured with p-nitrophenyl palmitate substrate following a previously published protocol (26). Enzyme activity values of PNLIP variants were normalized to that of the wild-type and expressed as percentage.

### Expression and purification of pancreatic lipase and chymotrypsins

For large-scale protein expression, HEK 293T cells were grown in a T75 flask and transiently transfected with PNLIP, CTRC, or CTRB1 plasmid DNAs using a previously described protocol (37). After 6 h of incubation, the cells were rinsed with PBS and incubated in 15 ml of Opti-MEM I medium at 37 °C in a standard tissue culture incubator. After 48 h of incubation, the PNLIP-containing conditioned medium was harvested. Fresh Opti-MEM was added to the flask again and collected after an additional 48 h of incubation. Target proteins were typically purified from the conditioned media of 10 flasks of transfected cells using a 5 ml HisPur nickel nitrilotriacetic acid (Ni-NTA) affinity cartridge (Thermo Scientific) attached to an Akta Purifier chromatography system as described previously (31). Aliquots of the eluted fractions were analyzed by 15% sodium dodecyl sulfate polyacrylamide gel electrophoresis (SDS-PAGE) and Coomassie staining. Fractions containing pure PNLIP protein were pooled and dialyzed against 3 × 1000 ml of 50 mM Tris-HCl buffer (pH 8.0) containing 100 mM NaCl using Spectra/Por Float-A-Lyzer Dialysis devices (Spectrum Labs) with molecular weight cut-off of 10 kDa. Fractions containing pure chymotrypsin were pooled and dialyzed against 3 × 1000 ml of 0.1 M Tris-HCl buffer (pH 8.0) supplemented with 150 mM NaCl. Protein samples were concentrated using Amicon Ultra centrifugal filters (Merck Millipore). The concentration of the PNLIP protein was determined from the absorbance values at 280 nm using a molar extinction coefficient of 61,600 M^-1^ cm^-1^. Chymotrypsins were activated with 5 nM cationic trypsin and their concentrations were determined by active site titration against ecotin inhibitor.

### Expression and purification of pancreatic trypsinogens

Trypsinogens were expressed in BL21(DE3) *E. coli*, isolated as cytosolic inclusion bodies, *in vitro* refolded, and purified with ecotin affinity chromatography according to a previously described protocol (38). Purified trypsinogen samples were activated with recombinant human enteropeptidase (Bio-Techne R&D Systems) and their concentrations were determined by active site titration against the inhibitor ecotin.

### SDS-PAGE and densitometry

Pancreatic lipase samples from conditioned media (200 μl) or proteolysis experiments (75 μl) were precipitated with 10% trichloroacetic acid (final concentration) and incubated for 5 min on ice. The PNLIP precipitate was pelleted by centrifugation at 17,000×*g* for 10 min in a tabletop microcentrifuge and solubilized in 15 μl of Laemmli sample buffer supplemented with 100 mM dithiothreitol. After heat denaturation at 95 °C for 5 min, the proteins were electrophoresed on 15% polyacrylamide mini gels followed by Coomassie Blue R250 staining. For certain experiments, PNLIP precipitates were solubilized in Laemmli sample buffer without the addition of dithiothreitol. Under these conditions, the protein samples were analyzed without prior heat denaturation. After destaining with 10% methanol and 10% acetic acid solution, the gels were visualized using an Azure 600 gel imaging device (Azure Biosystems). Quantitation of bands was performed with Quantity One 4.6.6 (Bio-Rad). Rectangles were placed around the bands and background subtraction was executed using isometric rectangles positioned within the corresponding lanes.

### Western blot

Total protein (10 μg) from the cell lysates was mixed with equal amounts of Laemmli buffer containing 100 mM dithiothreitol. After denaturation at 95 °C for 5 min, the samples were electrophoresed on a 15% polyacrylamide gel and transferred onto an Immobilon-P membrane (Merck Millipore). The membrane was blocked with 5% non-fat milk in PBS supplemented with 0.1% Tween 20. To detect PNLIP, an anti-His tag antibody conjugated to horseradish peroxidase (HRP) (Qiagen, catalog number 34460) was added at a dilution of 1:8000 for 1 h. GAPDH protein was detected using rabbit anti-GAPDH antibody (Sigma-Aldrich, catalog number G9545) at a dilution of 1:4000 for 1 h, followed by the addition of HRP-conjugated goat anti-rabbit IgG (Bio-Rad, catalog number 1706515) as a secondary antibody at a dilution of 1:4000 for 1 h. Protein bands were visualized using the SuperSignal West Pico Plus chemiluminescent substrate (Thermo Scientific) and Azure 600 gel imaging device.

### Model building

The structure of human PNLIP was retrieved from the Protein Data Bank database (PDB ID: 1N8S) (39). The colipase and any other moieties were removed from the structure. A structural model of mouse PNLIP was prepared by AlphaFold (40), through ColabFold (41) based on the amino acid sequence of C57BL/6J PNLIP without the signal sequence. The presence of disulfide bonds was evaluated by visual inspection of the structures. The Chimera (42) software was applied to determine the protonation states of histidine residues, and the PROPKA 3.1 (43, 44) software was used to determine the pKa values of titratable residues. The pH value of 8.0 was assumed when setting the protonation states. To create models of the I265R variants of both human and mouse PNLIP, the mutation was introduced using the Chimera (42) software and the Dynameomics rotamer library (45). The protonation states were determined using the same protocol as for the wild-type.

### Molecular dynamics simulation

The structures were prepared with the tleap script of AmberTools22 (46). Disulfide bonds were also added. A truncated octahedron simulation box with a minimal distance box parameter of 12 Å was applied, and TIP3P water (47) molecules were added to the systems. The ensembles were neutralized with the addition of sodium ions. The ff14SB (48) force field was applied to describe the protein atoms. The simulations were carried out with Amber22 (46), taking advantage of GPU acceleration (49, 50).

The systems were first minimized, applying the steepest descent method of 2500 steps, followed by a conjugate gradient method for a further 7500 steps. Positional restraints with 10 kcal mol^-1^ Å^-2^ were applied to the protein-heavy atoms. The systems were heated to 100 K (−173.15 °C) in a canonical NVT ensemble during a 250 ps simulation, followed by heating to 310.15 K (37 °C) in an isothermal-isobaric NPT ensemble, during a 250 ps simulation, using the same restraint. Langevin dynamics (51) and the Berendsen barostat (52) were employed to control temperature and pressure, respectively. To constrain bonds involving hydrogen atoms, we applied SHAKE (53). An additional minimization was carried out using the above mentioned steps with decreased positional restraints of 5 kcal mol^−1^ Å^−2^ on protein heavy atoms. Afterward, the above-described heating protocol was applied with reduced restraint. The systems were further equilibrated in an NPT ensemble at 310.15 K for six consecutive, 100 ps long simulations, in each, decreasing the positional restraint value by 1 kcal mol^−1^ Å^−2^ until the last equilibration that was run without restraints. The production run was carried out at 310.15 K for 300 ns with 2 fs timesteps. Sampling was done with a 50 ps time interval. Three independent replicas were run for each system.

### Simulation analysis

The analysis of the trajectories was carried out with the cpptraj program (54). The RMSF values were calculated for the Cα atoms of each residue. First, the N-terminal domains of the frames were aligned, and the RMSF values for the C-terminal domain residues were calculated. Next, the residues in the C-terminal domain were aligned and the RMSF values for the N-terminal domain were calculated. The ensembles were clustered with the average-linkage method, with an epsilon value of 2.0 Å. The mask for the clustering included the Cα atoms of each residue. The clustering was performed only for every fifth frame by setting the sieve value to 5. After the clustering, all other frames were added to the clusters. The hydrogen bond analysis was carried out for each residue with the hbond command. Both the acceptor and the donor interactions were evaluated. Hydrogen bonds present in at least 10% of the trajectories were considered as existing interactions. The average hydrogen bond number per residue was determined by adding up the hydrogen bond counts for each residue over all frames and then averaging these sums by the number of frames. Residues interacting with the Ile265 in the wild-type PNLIP and Arg265 in the I265R variant were determined using LigPlot+ (55).

### Data analysis

Statistical analyses relative to wild-type values were performed with a one-way analysis of variance technique using R (version 4.3.2) through RStudio (56, 57). Tukey’s Honest Significant Difference *post hoc* test was applied to determine comparisons between group means. *p*-values ≤ 0.05 were considered significant.

## Data availability

All data are contained within the manuscript.

## Supporting information

This article contains supporting information.

## Conflict of interest

The authors declare that they have no conflicts of interest with the contents of this article.
