# Supplementary material for: Modeling protease-sensitive human pancreatic lipase mutations in the mouse ortholog
Source: J Biol Chem. 2024 Sep 10;300(10):107763. doi: 10.1016/j.jbc.2024.107763 (PMC11489347; doi:10.1016/j.jbc.2024.107763)
Supplement: Supporting Table and Figures [file mmc1.pdf]

Supporting information

Modeling protease-sensitive human pancreatic lipase mutations in the mouse ortholog

Gyula Hoffka, Samara Mhana, Marcell Vas, Vanda Toldi, János András Mótyán, József Tőzsér, András Szabó

Table of Contents

Figure S-1. Effect of colipase on the degradation of mouse I265R PNLIP variant by mouse cationic T7 trypsin

Figure S-2. Degradation of mouse PNLIP by human cationic trypsin

Table S1. Presence of relevant hydrogen bonds in the 341-355 region of mouse PNLIP

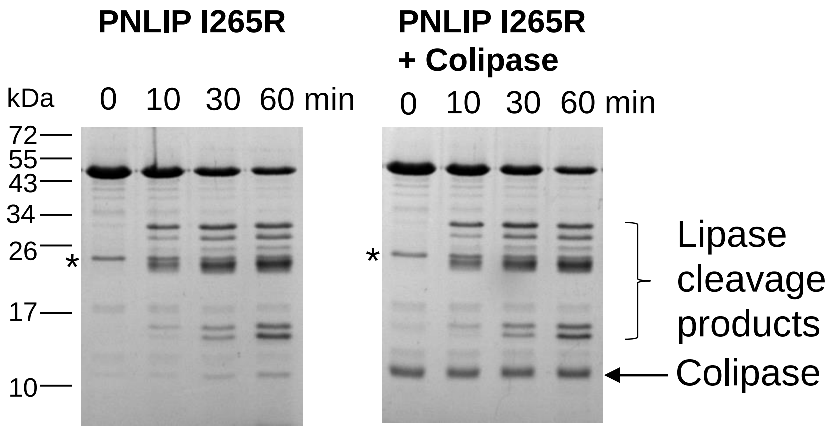

Figure S-1. Effect of colipase on the degradation of mouse I265R PNLIP variant by mouse cationic T7 trypsin. Purified PNLIP variant at a final concentration of 2 μM was incubated at 37 °C with 200 nM human cationic trypsin in 0.1 M Tris-HCl (pH 8.0), 50 mM NaCl, and 1 mM $CaCl_2$ (final concentrations) in the absence and presence of 2 μM colipase. At the indicated times, 75 μL aliquots were precipitated with 10% trichloroacetic acid (final concentration) and analyzed by reducing SDS-PAGE and Coomassie Blue staining. The asterisk indicates the trypsin band.

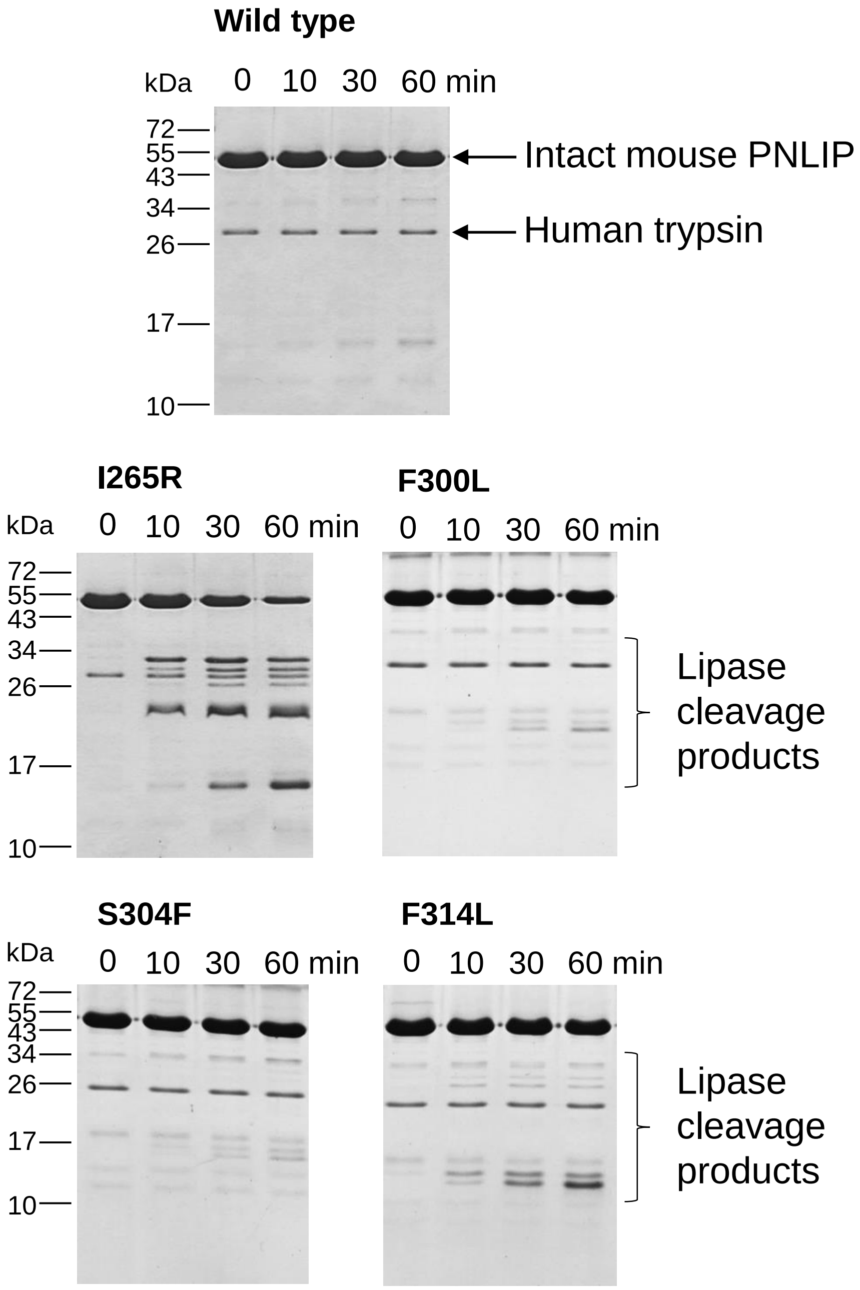

Figure S-2. Degradation of mouse PNLIP by human cationic trypsin. Purified wild-type and mutant PNLIP proteins at a final concentration of 2 μM were incubated at 37 °C with 200 nM human cationic trypsin in 0.1 M Tris-HCl (pH 8.0), 50 mM NaCl, and 1 mM $CaCl_2$ (final concentrations). At the indicated times, 75 μL aliquots were precipitated with 10% trichloroacetic acid (final concentration) and analyzed by reducing SDS-PAGE and Coomassie Blue staining. Representative gels of two experiments are shown.

Table S1. Presence of relevant hydrogen bonds in the 341-355 region of mouse PNLIP. The average number of hydrogen bond interactions of the residues labeled in red is shown in Figure 9.

Residue (**Mouse**/*Human*)	$N_{H\text{-}bond}$ (Mouse)	$N_{H\text{-}bond}$ (Human)
**Thr341**/*Lys341*	1.07	0.58
**Phe342**	1.26	1.36
**Tyr343**	1.32	1.30
**Leu344**	0.93	0.89
**Asn345**/*Asp345*	1.72	1.59
**Thr346**	0.87	1.05
**Gly347**	0.61	0.64
**Asp348**	1.01	0.59
**Lys349**/*Ala349*	0.68	0.42
**Ser350**	0.06	0.20
**Asn351**	0.85	1.07
**Phe352**	0.98	0.60
**Ala353**	0.91	0.33
**Arg354**	1.85	2.59
**Trp355**	1.78	2.06
